# Molecular mechanisms of dose-dependent regulation of hepatic lipid metabolism by BaP through modulation of AhR binding to XRE1 or XRE3

**DOI:** 10.3389/fphar.2025.1595566

**Published:** 2025-07-25

**Authors:** Mengdi Zhang, Xiaoli Lv, Chaojie Wang, Lei Wang, Han Wang, Xue Wang, Yulu Du, Jun Li, Xiuli Han, Lei Fan, Yuxia Hu, Tuya Bai, Weizhong Huangfu, Fuhou Chang

**Affiliations:** ^1^ College of Pharmacy, Inner Mongolia Medical University, Hohhot, China; ^2^ Inner Mongolia Autonomous Region Engineering Research Center of New Pharmaceutical Screening, Inner Mongolia Medical University, Hohhot, China; ^3^ Center for New Drug Safety Evaluation and Research, Inner Mongolia Medical University, Hohhot, China; ^4^ Affiliated Hospital of Inner Mongolia Medical University, Hohhot, China

**Keywords:** benzo[a]pyren, fibroblast growth factor 21, aryl hydrocarbon receptor, xenobiotic response element 1, xenobiotic response element 3, hepatic lipid metabolism

## Abstract

Benzo[a]pyrene (BaP), a polycyclic aromatic hydrocarbon and a potent environmental pollutant, has been implicated in the dysregulation of lipid metabolism and metabolic diseases, warranting investigation into its effects on liver functions, particularly regarding fibroblast growth factor 21 (FGF21) mediated pathways. This study aimed to elucidate the effects of BaP on liver lipid metabolism and FGF21 expression via the aryl hydrocarbon receptor (AhR), with a focus on the regulatory interactions between BaP and xenobiotic response elements (XRE) within the promoter region of FGF21. Utilizing HepG2 cells, lipid accumulation was assessed through Oil Red O and Nile Red staining, while the expression of FGF21 protein was quantified by Western blotting and immunofluorescence techniques. Additionally, various truncated plasmids of the FGF21 promoter were synthesized for a dual-luciferase reporter assay to determine the relative luciferase activity and the modulation of FGF21 expression by BaP. The results revealed dose-dependent effects of BaP on lipid metabolism; specifically, low concentrations of BaP upregulated FGF21 expression by enhancing promoter activity in regions containing the XRE1 sequence, whereas higher BaP concentrations downregulated FGF21 expression via inhibition of promoter activity in regions with the XRE3 sequence. In conclusion, low doses of BaP facilitate AhR binding to XRE1, promoting FGF21 expression, while high doses disrupt this interaction through XRE3, culminating in decreased expression levels. These findings suggest a nuanced role of BaP in lipid metabolism regulation, with potential implications for understanding metabolic disorders associated with environmental pollutants. The study elucidates the relationship between AhR and FGF-21, providing an experimental basis for the search of new targets for the prevention and treatment of nonalcoholic fatty liver disease (NAFLD).

## Introduction

The liver is a key metabolic organ in the human body, primarily responsible for lipid metabolism. Dietary fats are synthesized and stored in the liver and are subsequently broken down into glycerol and fatty acids to provide energy when needed. When lipid metabolism is disrupted, it increases the burden on the liver, potentially leading to conditions such as fatty liver, hepatitis, cirrhosis, or even hepatocellular carcinoma. Among these conditions, non-alcoholic fatty liver disease (NAFLD) is the most common type. NAFLD is a metabolic disorder characterized by excessive lipid accumulation in the liver due to abnormal lipid metabolism (Stone et al., 2014; Friedman et al., 2018), which may progress to hepatitis or non-alcoholic steatohepatitis (NASH). NASH can further lead to liver fibrosis, cirrhosis, and may even cause liver failure or hepatocellular carcinoma (Zhou et al., 2020). Environmental factors and unhealthy dietary habits are the primary risk factors for NAFLD. Therefore, this study aims to explore the mechanisms underlying hepatic lipid metabolism disorders from the perspective of environmental pollutants.

Benzo[a]pyrene (BaP) is a polycyclic aromatic hydrocarbon (PAH) compound that is widely found in cigarette smoke, charred food, and industrial emissions. BaP is chemically stable, highly lipophilic, and easily soluble in nonpolar solvents, allowing it to accumulate in adipose tissue and the liver after entering the human body through the respiratory and digestive systems. Prolonged low-dose exposure to BaP may lead to its accumulation in the body, triggering a range of toxic effects that can damage tissues and organs (Marzooghi and Di Toro, 2017). In daily life, the primary source of BaP is food, and its metabolism in the liver may impair liver function (Srogi, 2007). Epidemiological studies have shown that exposure to BaP and other PAHs (such as TCDD) is associated with dyslipidemia, impaired glucose metabolism, and altered liver function, potentially contributing to the development of NAFLD (Kumar et al., 2014; Taylor et al., 2013; Casals-Casas and Desvergne, 2011; Grün and Blumberg, 2009). Studies have demonstrated that mice exposed to PAHs show increased expression of hepatic cytochrome P450 enzymes, accompanied by liver tissue damage and the accumulation of phospholipids and triglycerides, which may lead to NAFLD, liver cell membrane damage, inflammation, and disruption of signaling pathways (Li et al., 2020; Guo et al., 2018). In this study, HepG2 cells were treated with appropriate doses of BaP to simulate its metabolic processes in the liver, aiming to investigate the effects of BaP on hepatic lipid metabolism.

The aryl hydrocarbon receptor (AhR) is a nuclear transcription factor that is activated upon binding to endogenous or exogenous ligands and subsequently translocates into the nucleus to form specific nuclear protein complexes, thereby exerting its function (Bersten et al., 2013). PAHs (such as BaP and TCDD) are classical exogenous AhR ligands capable of inducing the expression of cytochrome P450 enzymes through the AhR-XRE-dependent pathway. In the absence of ligands, AhR exists in an inactive state by forming a multiprotein complex with heat shock protein 90 (HSP90), hepatitis B virus X-associated protein (XAP), and p23, with its nuclear translocation system being suppressed. Upon ligand binding, such as with BaP, AhR forms a complex with the aryl hydrocarbon receptor nuclear translocator (ARNT), while HSP90 dissociates from the complex, leading to the formation of an AhR-ARNT complex, which subsequently activates the nuclear localization system of AhR. After translocation into the nucleus, the AhR-ARNT complex binds to XRE within the promoter regions of target genes, thereby regulating their transcription and expression (Larigot et al., 2018). BaP exerts its toxic effects by activating the AhR signaling pathway in the liver. Therefore, in this study, the AhR inhibitor CH223191 was used to investigate whether the effects of BaP on hepatic lipid metabolism are dependent on AhR activation.

FGF21 is a novel lipid metabolism regulator that primarily acts as a stress sensor in the liver and other endocrine organs, playing a crucial role in lipid metabolic regulation. Moreover, FGF21 serves as a diagnostic biomarker in metabolic diseases related to glucose and lipid metabolism, with elevated serum FGF21 levels observed in early-stage NAFLD patients. Studies have shown that AhR is associated with the transcriptional regulation of FGF21. Girer et al. (2019) reported that the FGF21 promoter contains three XRE domains (XRE1, XRE2, and XRE3), and in the livers of mice treated with TCDD, AhR binding decreased at XRE3, increased at XRE1, and remained unchanged at XRE2. These findings suggest that AhR binding at XRE1 promoters FGF21 transcription, while binding at XRE3 inhibits its transcription. To date, there is no literature reporting how BaP-activated AhR interacts with XRE domains or its effects on FGF21 expression. Our previous studies have shown that short-term exposure to low doses of BaP increases cytochrome P450 enzyme expression in mouse liver and elevates FGF21 levels. However, this increase in FGF21 expression was suppressed by the addition of an AhR inhibitor, indicating that low-dose BaP influences FGF21 expression through AhR (Su, 2023). Therefore, in this study, we will employ a dual-luciferase reporter gene assay to investigate BaP’s regulatory effect on AhR binding to XREs within the FGF21 promoter region and examine BaP’s impact on the expression of FGF21 and related factors, providing new insights for the prevention and treatment of glucose and lipid metabolism disorders induced by BaP.

## Materials and methods

### Experimental materials

BaP was procured from Sigma (Beijing, China). BCA protein quantitative analysis kit was procured fromThermo Fisher Scientific (Shanghai, China). FGF-21 antibody and PPARγ antibody were procured from ABclonal(Wuhan, China). PPARα, CPT-1α, PGC1α, C/EBPα and β-actin antibody were procured from Abcam(Shanghai, China). Dylight 800 Goat Anti-Rabbit igG was procured from Abbkine(Wuhan, China). Modified Oil Red O Staining Kit and Lipid Droplet Red Fluorescence Detection Kit were procured from Beyotime(Shanfhai, China). Sodium dodecyl sulfonate, Glycine and Tris-base were procured from Solarbio(Beijing, China). 5x loading buffer (SDS-PAGE) was procured from Applygen(Shanghai, China). pGL4, pGL4-FGF21-promoter-1, pGL4-FGF21-promoter-2, pGL4-FGF21-promoter-3, and pRL-TK were all purchased from GenePharma Company(Shanghai, China).

### Cell culture

HepG2 cells were cultured in DMEM medium containing 10% fetal bovine serum (FBS) and 1% penicillin-streptomycin. Cells were maintained in an incubator at 37°C with 5% CO_2_. After thawing, cells were centrifuged at 1,500 rpm for 5 min and resuspended in fresh medium. When the cell confluence reached 80%–90%, the cells were digested with 0.25% trypsin and passaged at a 1:2 ratio into new culture flasks.

### Cell treatment

Healthy HepG2 cells were selected and prepared as a cell suspension according to the procedure in 2.1. The cell suspension was evenly seeded into 6-well plates, with each well containing 2 mL of complete culture medium, and cultured for 24 h. The following groups were established: Control, 15 μmol/L BaP, 30 μmol/L BaP, and 60 μmol/L BaP groups. Cells in each group were treated with the corresponding concentrations of BaP and incubated for 24 h before subsequent experiments. Another set of groups was established: Control, 30 μmol/L BaP, and 30 μmol/L BaP + 1 μmol/L CH223191. In the 30 μmol/L BaP + 1 μmol/L CH223191 group, cells were pretreated with 1 μmol/L CH223191 for 12 h before adding 30 μmol/L BaP and then incubated for another 24 h (Lou et al., 2022; Petroff et al., 2011). The other groups were treated with the corresponding drugs for 24 h.

### MTT assay

MTT reagent was prepared to the required concentration (5 mg/L) according to the kit instructions. A cell suspension was prepared following the protocol in 2.1. A suitable volume of the cell suspension was added to a hemocytometer for cell counting, and the cell suspension was diluted to a concentration of 5 × 10^4^ to 8 × 10^4^ cells/mL using complete medium based on the counting results. BaP concentrations were set at 0, 1, 2, 4, 8, 16, 32, 64, 128, 256 and 512 μmol/L, with control and apoptosis wells. Each group was prepared in six replicates and treated with the corresponding drugs for 24 h. Then, 5 mg/mL MTT solution was added to each well, mixed thoroughly, and incubated for another 4 h. Next, 100 μL of DMSO solution was added, and the plates were shaken gently until the formazan crystals dissolved completely. The absorbance was measured to calculate cell viability. A concentration-growth activity curve was plotted to determine the appropriate BaP concentration for subsequent experiments.

### Nile red fluorescence staining

A 12-well plate was prepared by adding 100 μL of PBS to each well, followed by placing cell coverslips evenly in the wells. BaP concentrations were set at 0, 15, 30, and 60 μmol/L, and cells were treated with the respective drugs and incubated for 24 h. After incubation, the cells were fixed with 0.5 mL of paraformaldehyde per well for 30 min, and then the paraformaldehyde was removed. The cells were washed three times with PBS. Nile Red staining solution was prepared at a dilution ratio of 1:200 (Nile Red stock solution: diluent). For each well, 100–300 μL of staining solution was added, and the cells were stained in the dark for 20 min. The coverslips were then placed on glass slides and observed using a digital pathology scanning system. Images were captured and analyzed using ImageJ for data visualization.

### Oil red O staining

Cells prepared according to the protocol in 2.1 were washed with 1 mL of PBS per well (in a 6-well plate) three times. Next, 1 mL of paraformaldehyde was added to each well for fixation for 30 min. After removing the paraformaldehyde, the cells were washed three times with PBS. Then, 400 μL of Oil Red O staining solution was added to each well for 20 min. After staining, the cells were thoroughly washed three times using the washing solution, followed by three additional PBS washes (1 min each). Images were captured under an optical microscope for further analysis.

### Western blotting (WB)

Protein samples were prepared by boiling in a solution of protein loading buffer [100 mM Tris (pH 6.8), 25% glycerol, 2% SDS, 0.01% bromophenol blue, 10% 2-mercaptoethanol] at a 1:1 volume ratio for 15 min. The protein samples (20–50 μg) were separated by electrophoresis on a Tris-HCl PAGE gel and transferred onto a PVDF membrane using wet transfer. The membrane was blocked with Tris-buffered saline containing 0.1% Tween 20% and 5% non-fat milk. The membrane was then incubated with specific primary antibodies, followed by secondary antibody incubation, allowing for hybridization. The bands were detected using an Odyssey infrared imaging system (Hong Kong Gene Company). Band intensities were quantified using ImageJ software, and the values were normalized to the corresponding protein levels in each sample.

### Immunofluorescence

HepG2 cells were seeded onto coverslips placed in a 12-well plate. When the cell confluence reached approximately 70%, staining was initiated. The cells were fixed with 4% paraformaldehyde [4°C] for 10 min, followed by three PBS washes. The cells were then blocked with 5% BSA for 50 min and incubated with the primary antibody (1:200 dilution) at 4°C for 24 h. After incubation, the cells were washed three times with PBS in the dark. The secondary antibody diluted in PBS was added and incubated at room temperature for 40 min in the dark. The cells were washed four times with PBS and stained with DAPI staining solution in the dark for 10 min. Excess DAPI was removed as much as possible from the coverslips. The cells were washed once with PBS to eliminate residual DAPI. A total of 20 μL of anti-fade mounting medium (purchased from Beyotime Biotechnology) was added to each coverslip in the dark. Images were acquired using a digital pathology scanning system.

### Construction of truncated plasmids containing different FGF21 promoter regions

**FIGURE 1:**
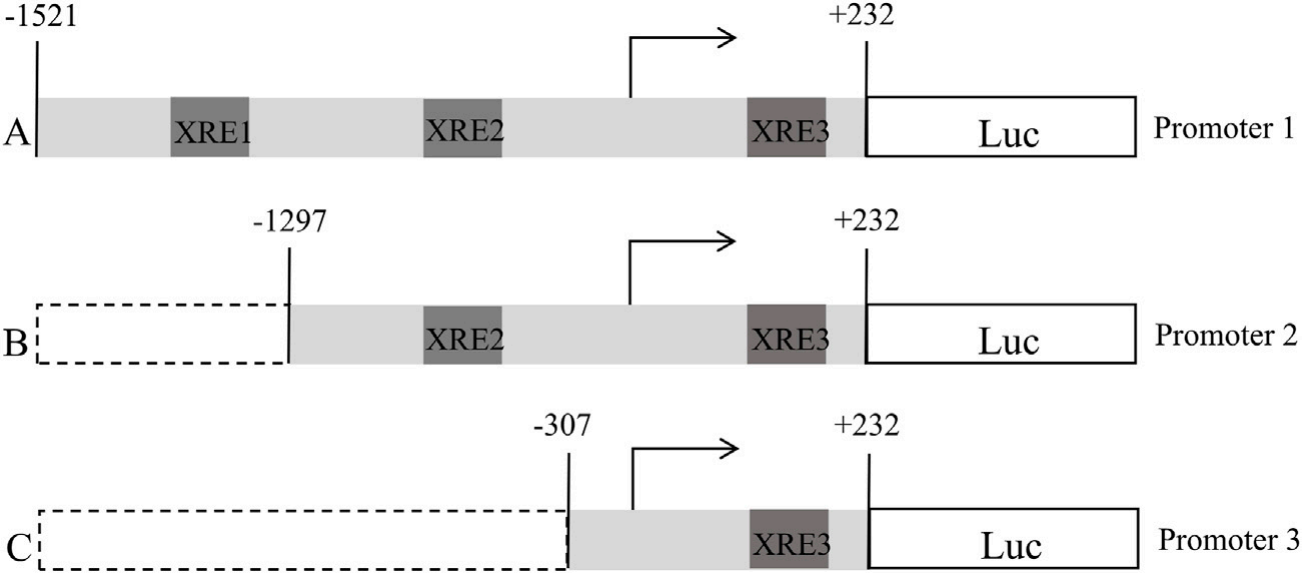
Schematic representation of the construction of a truncated plasmid containing an XRE fragment in the promoter region of FGF21 (A:pGL4-FGF21-promoter-1, B:pGL4-FGF21-promoter-2, C:pGL4-FGF21-promoter-3).

Based on the FGF21 promoter region sequence obtained from UCSC, three XRE fragments were predicted. According to the locations of these three XRE fragments, three plasmids were designed, as shown in Figure 1. Based on the FGF21 promoter region sequence obtained from UCSC, three XRE(5′-GCGTG-3′) fragments were predicted, namely XRE1 (located at nucleotide positions −1,479 to −1,474), XRE2 (−1,235 to −1,230), and XRE3 (+190 to +195). Based on the positions of these three XRE fragments, three plasmids were designed using pGL4 as the blank plasmid, with Promoter-1 being the full-length plasmid, and Promoter-2 and Promoter-3 being truncated plasmids, as shown in Figure 1. After plasmid construction, sequencing was performed to verify their correctness. pRL-TK serves as an internal control plasmid. Detection and data analysis were conducted according to the instructions of the Dual Luciferase Reporter Gene Assay Kit.

### Statistical analysis

ImageJ software was used for image visualization and analysis. GraphPad Prism 7 was employed for statistical analysis, where the t-test was used for comparisons between two groups, and one-way ANOVA was used for comparisons among multiple groups. Data were expressed as mean ± standard deviation (x̄ ± s), and differences were considered statistically significant at *P* < 0.05.

## Results

### Effects of different concentrations of BaP on cell viability

**FIGURE 2:**
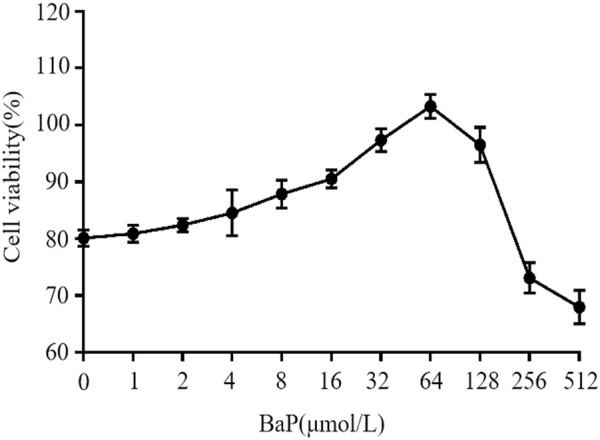
Effect of BaP on cell growth activity.

The effects of different concentrations of BaP on the viability of HepG2 cells are shown in Figure 2. BaP concentrations ranging from 0 to 64 μmol/L did not exhibit any inhibitory effect on cell viability. However, when the BaP concentration exceeded 64 μmol/L, a significant inhibitory effect on cell viability was observed. Therefore, the concentrations of 0, 15, 30, and 60 μmol/L BaP were selected for subsequent experiments.

### Effects of different BaP concentrations on lipid fluorescence in HepG2 cells

**FIGURE 3:**
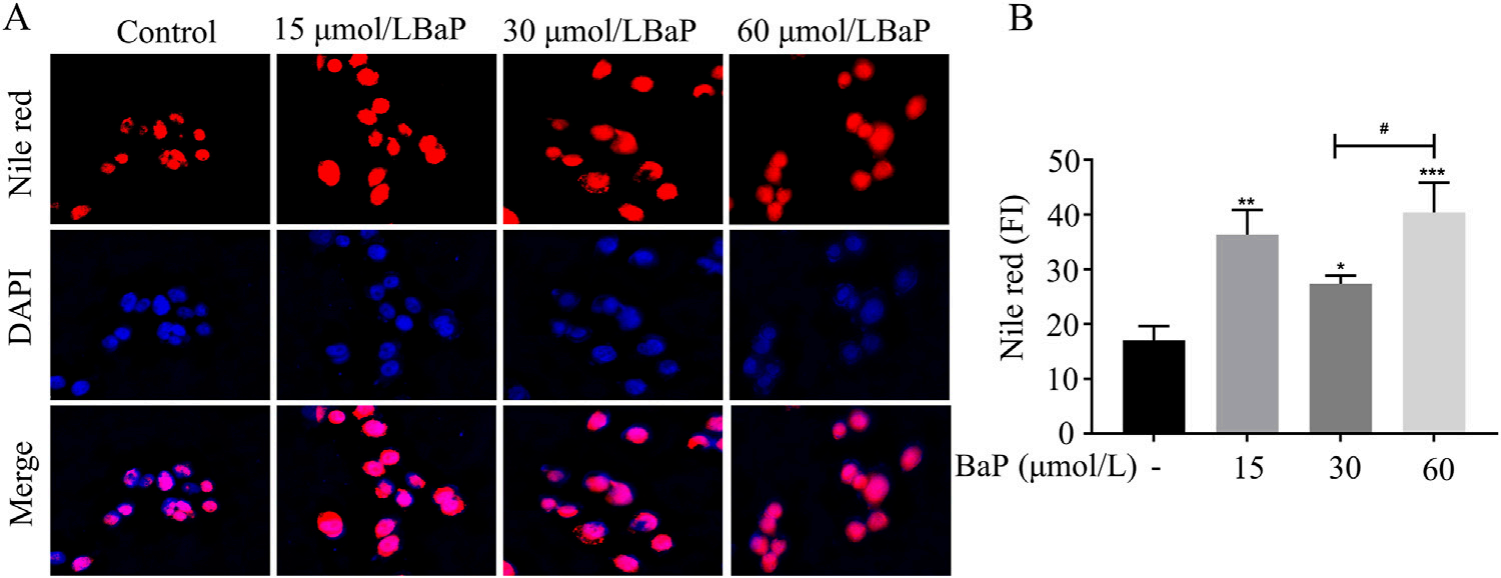
Effect of different concentrations of BaP on lipid fluorescence in HepG2 cells (**(A)** lipid fluorescence plot, **(B)** lipid fluorescence intensity statistic, ^*^ *P* < 0.05, ^**^ *P* < 0.01, ^***^ *P* < 0.001 compared with Control group; ^#^ *P* < 0.05 compared with 30 μmol/L BaP group, n = 3).

After treatment with different concentrations of BaP, the lipid fluorescence intensity in HepG2 cells is shown in Figure 3A. Nile Red staining revealed a significant increase in lipid fluorescence intensity in cells treated with 15, 30, and 60 μmol/L BaP compared to the control group (*P* < 0.05). Notably, the lipid fluorescence intensity was significantly higher in the 60 μmol/L BaP group than in the 30 μmol/L BaP group (*P* < 0.05, Figure 3B). These findings indicate a dose-dependent increase in intracellular lipid accumulation with rising BaP concentrations, reflecting the impact of BaP on lipid metabolism.

### Effects of different BaP concentrations on lipid accumulation in HepG2 cells

**FIGURE 4:**
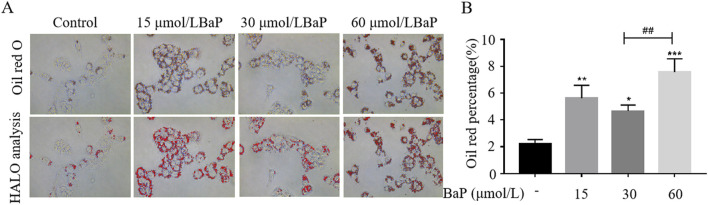
Effects of different concentrations of BaP on lipid content in HepG2 cells (**(A)** Oil red O staining plot, **(B)** HALO analysis statistic, ^*^ *P* < 0.05, ^**^ *P* < 0.01, ^***^ *P* < 0.001, compared to Control group; ^##^ *P* < 0.01 compared to 30 μmol/L BaP group, n = 3).

To further investigate the effects of different BaP concentrations on lipid metabolism in HepG2 cells, Oil Red O staining was used to observe lipid content in HepG2 cells (Figure 4A). As shown in Figure 4B, compared with the control group, lipid content in HepG2 cells significantly increased after treatment with 15, 30, and 60 μmol/L BaP (*P* < 0.05). Furthermore, lipid accumulation in the 60 μmol/L BaP group was significantly higher than that in the 30 μmol/L BaP group (*P* < 0.01). These results indicate that BaP promotes lipid accumulation in HepG2 cells, with 60 μmol/L BaP inducing the most pronounced lipid accumulation, while 30 μmol/L BaP had a relatively smaller impact on lipid content.

### Effects of different BaP concentrations on FGF21 expression in HepG2 cells

**FIGURE 5:**
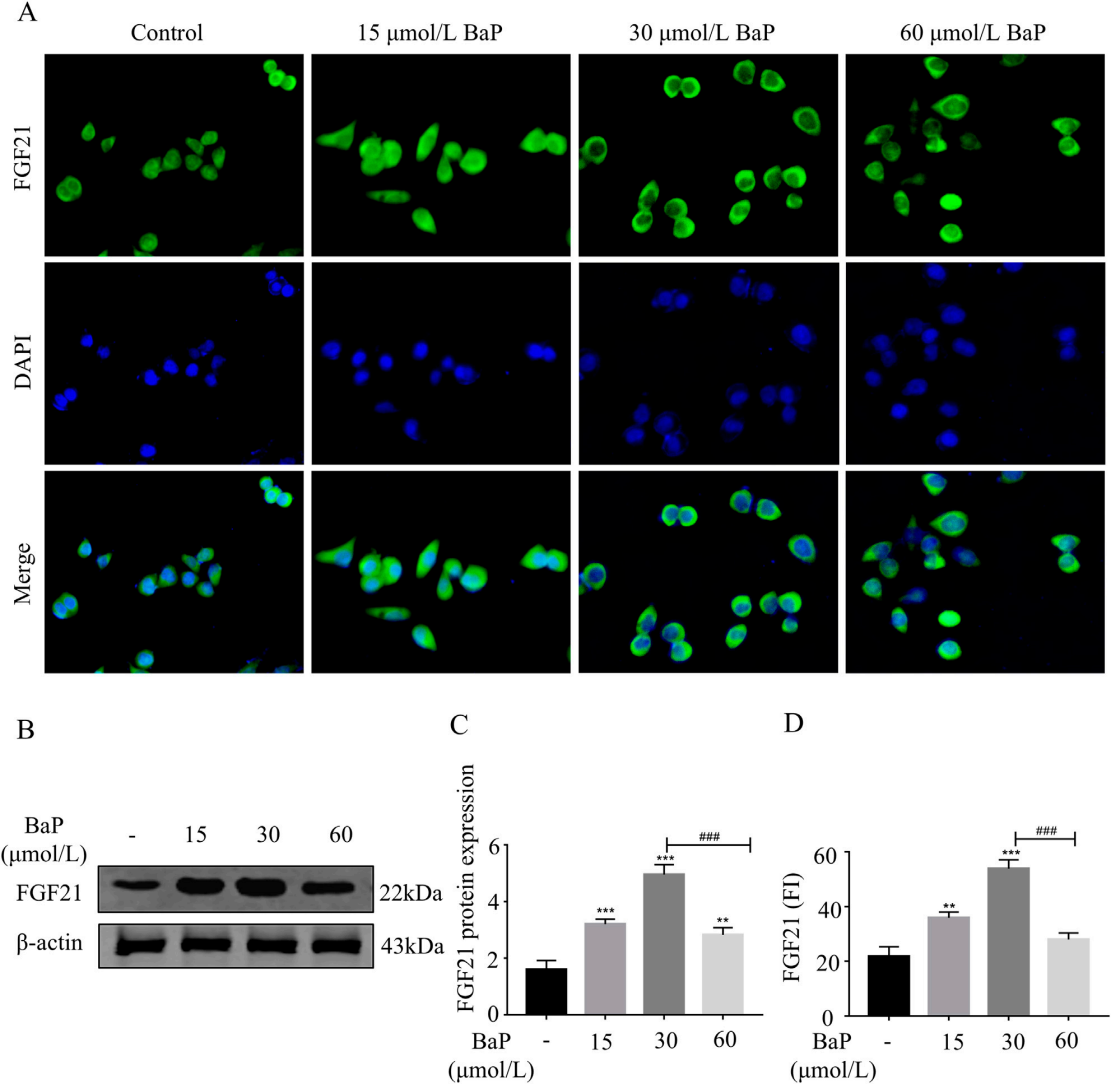
Effects of different concentrations of BaP on FGF21 expression in HepG2 cells (**(A)** Protein fluorogram, **(B)** protein bar graph, **(C)** protein expression statistic, **(D)** Fluorescence Intensity Statistics ^**^ *P* < 0.01, ^***^ *P* < 0.001 compared with Control group; ^###^ *P* < 0.001 compared with 30 μmol/L BaP group, n = 3).

To further clarify the effects of different BaP concentrations on FGF21 protein expression, immunofluorescence staining was used to assess the expression of FGF21 protein in BaP-treated HepG2 cells. As shown in Figures 5A,D, the fluorescence intensity of FGF21 protein increased in all BaP-treated groups (15, 30, and 60 μmol/L) compared to the control group, with the strongest fluorescence observed in the 30 μmol/L BaP group (*P* < 0.001). Among the treatment groups, the 60 μmol/L BaP group exhibited relatively weaker FGF21 protein fluorescence intensity. The Western blot results are shown in Figures 5B,C. After treatment with different concentrations of BaP, the expression of FGF21 protein in HepG2 cells was upregulated (P < 0.01), but the extent of upregulation varied. Compared to the 60 μmol/L BaP group, the 30 μmol/L BaP group showed a more significant upregulation of FGF21 expression (*P* < 0.001). The immunofluorescence results were consistent with the Western blot findings, suggesting that low and high doses of BaP may regulate FGF21 expression through different mechanisms.

### Effects of BaP on lipid fluorescence in HepG2 cells after AhR inhibition

**FIGURE 6:**
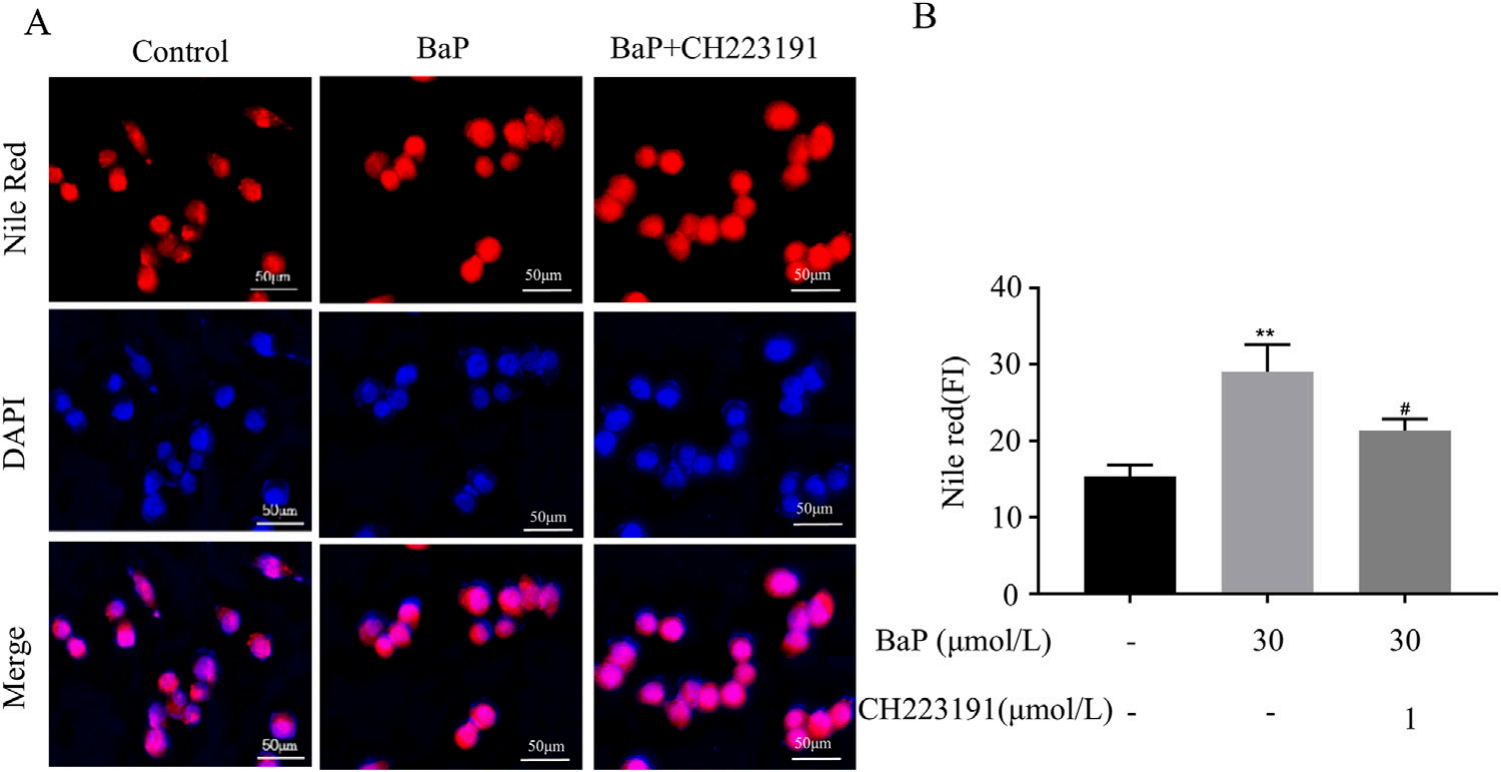
Effect of BaP action on lipid fluorescence in HepG2 cells 24 after inhibition of AhR (Concentrations of BaP and CH223191 were 30 μmol/L and 1 μmol/L, respectively. **(A)** Lipid fluorescence plots, **(B)** Lipid fluorescence intensity statistic plots, ^**^ *P* < 0.01 compared to Control group; ^#^ *P* < 0.05 compared to BaP group, n = 3).

HepG2 cells were treated with CH223191 and BaP at the corresponding concentrations, and the cells in each group were stained with Nile Red dye for fluorescence analysis. Figure 6A illustrates representative images of cells treated with control, 30 μmol/L BaP, and 30 μmol/L BaP + 1 μmol/L CH223191. The Nile Red staining (red) highlights lipid droplets, while DAPI (blue) stains nuclei. Merged images show the co-localization of lipid droplets within the cellular environment. Figure 6B provides a quantitative analysis of Nile Red fluorescence intensity across the three groups. Compared to the control group, BaP treatment significantly increased lipid fluorescence intensity (*P* < 0.01). However, when cells were co-treated with BaP and CH223191, the fluorescence intensity was significantly reduced compared to BaP alone (*P* < 0.05), indicating that CH223191 effectively inhibits BaP-induced lipid accumulation. These results demonstrate that BaP increases lipid accumulation in HepG2 cells through AhR.

### Effects of BaP on FGF21 protein expression in HepG2 cells after AhR inhibition

**FIGURE 7:**
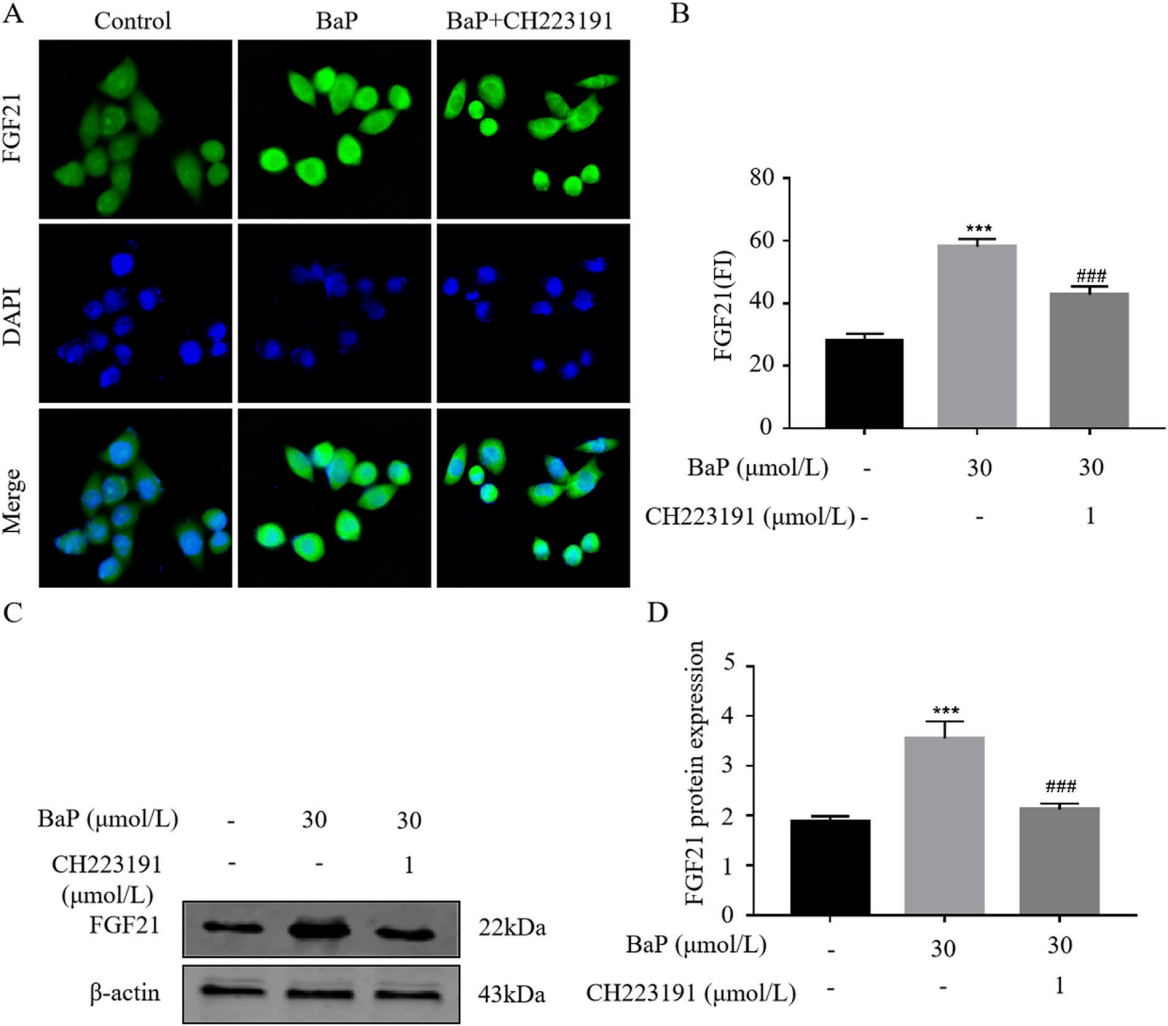
Effect of BaP action on FGF21 expression in HepG2 cells 24 after inhibition of AhR (**(A)** Protein fluorogram, **(B)** protein fluorescence statistics plot, **(C)** Protein bar graph, **(D)** Protein expression statistic, ^***^ *P* < 0.001 compared with Control group; ^##^ *P* < 0.01, ^###^ *P* < 0.001 compared with 30 μmol/L BaP group, n = 3).

To verify whether BaP activates FGF21 expression via the AhR pathway, HepG2 cells were treated with BaP and the AhR inhibitor CH223191, and relative protein expression levels were measured. The fluorescence images obtained from the digital pathology scanning system revealed clear cell morphology in all groups (Figure 7A). Consistent with the Western blot results, the fluorescence intensity of FGF21 protein significantly increased after treatment with 30 μmol/L BaP compared to the control group (*P* < 0.001), while co-treatment with 1 μmol/L CH223191 resulted in a marked reduction in FGF21 fluorescence intensity (*P* < 0.001, Figures 7B). As shown in Figures 7C,D, compared with the control group, FGF21 expression in HepG2 cells was significantly increased after treatment with 30 μmol/L BaP alone (*P* < 0.001). Furthermore, compared to the 30 μmol/L BaP group, FGF21 protein expression was significantly reduced in the 30 μmol/L BaP + 1 μmol/L CH223191 group (*P* < 0.01).

### Transcriptional activity of the FGF21 gene promoter region

**FIGURE 8:**
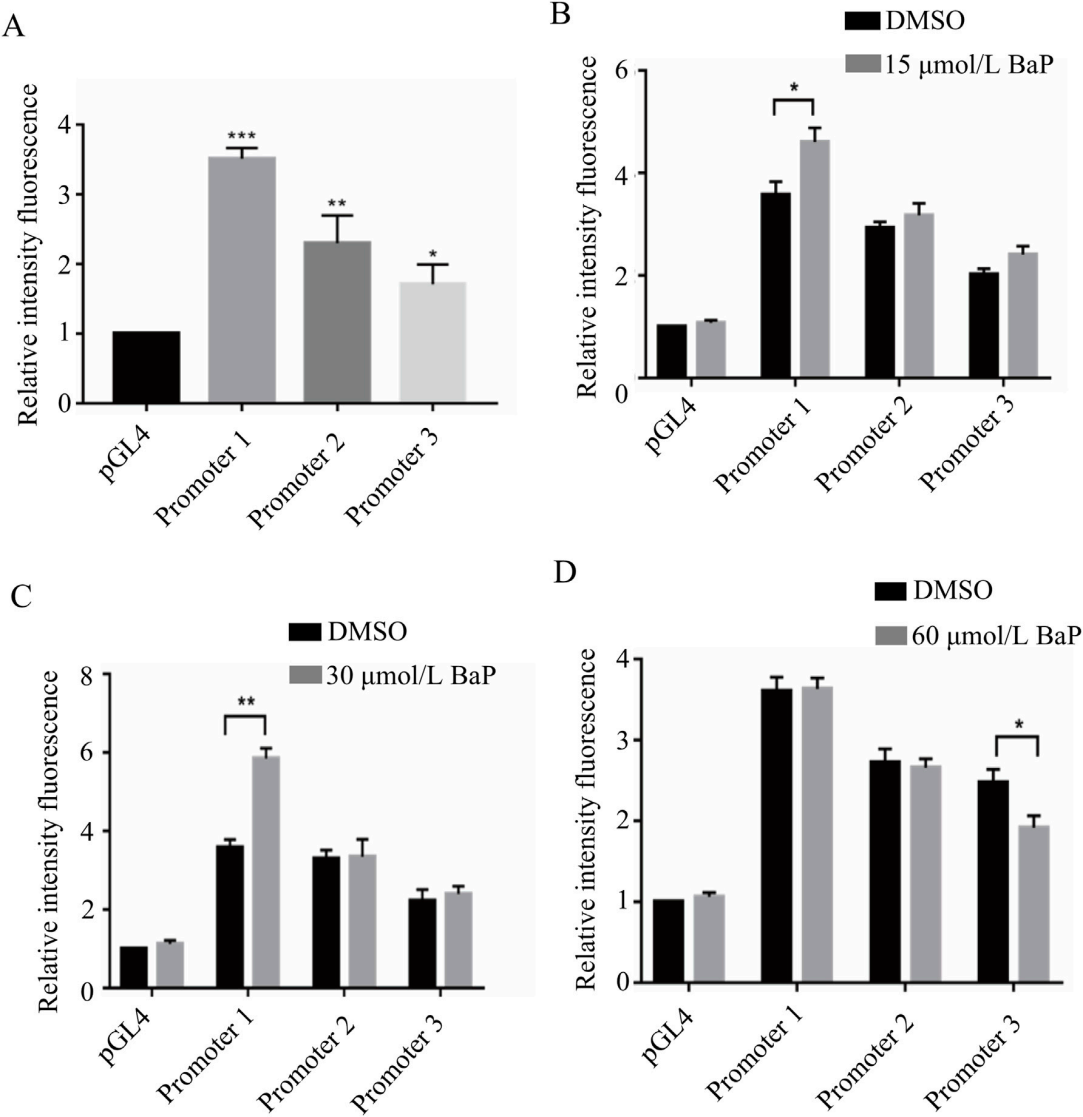
Transcriptional activity of fragments of the promoter region of the FGF21 gene by different concentrations of BaP (**(A)** Activity of transfected FGF21 gene promoter fragments in HepG2 cells, **(B)** Transcriptional activity of 15 μmol/L BaP on the promoter region fragment of FGF21 gene, **(C)** Transcriptional activity of 30 μmol/L BaP on the promoter region fragment of FGF21 gene, **(D)** Transcriptional activity of 60 μmol/L BaP on the promoter region fragment of FGF21 gene).

The results of the dual-luciferase reporter assay are shown in the figure. Compared to the pGL4-Basic vector, the relative luciferase activity in HepG2 cells transfected with the promoter1, promoter2, and promoter3 recombinant plasmids was significantly upregulated, with statistically significant differences (*P* < 0.05). Among them, the HepG2 cells transfected with the promoter1 recombinant plasmid showed the highest relative luciferase activity (*P* < 0.001, Figure 8A). After transfecting different recombinant plasmids into HepG2 cells and treating them with 15 μmol/L, 30 μmol/L, and 60 μmol/L BaP, the relative luciferase activity of each group is shown in Figures 8B–D, respectively.

### Effects of different BaP concentrations on FGF21 protein expression in HepG2 cells transfected with recombinant plasmids

**FIGURE 9:**
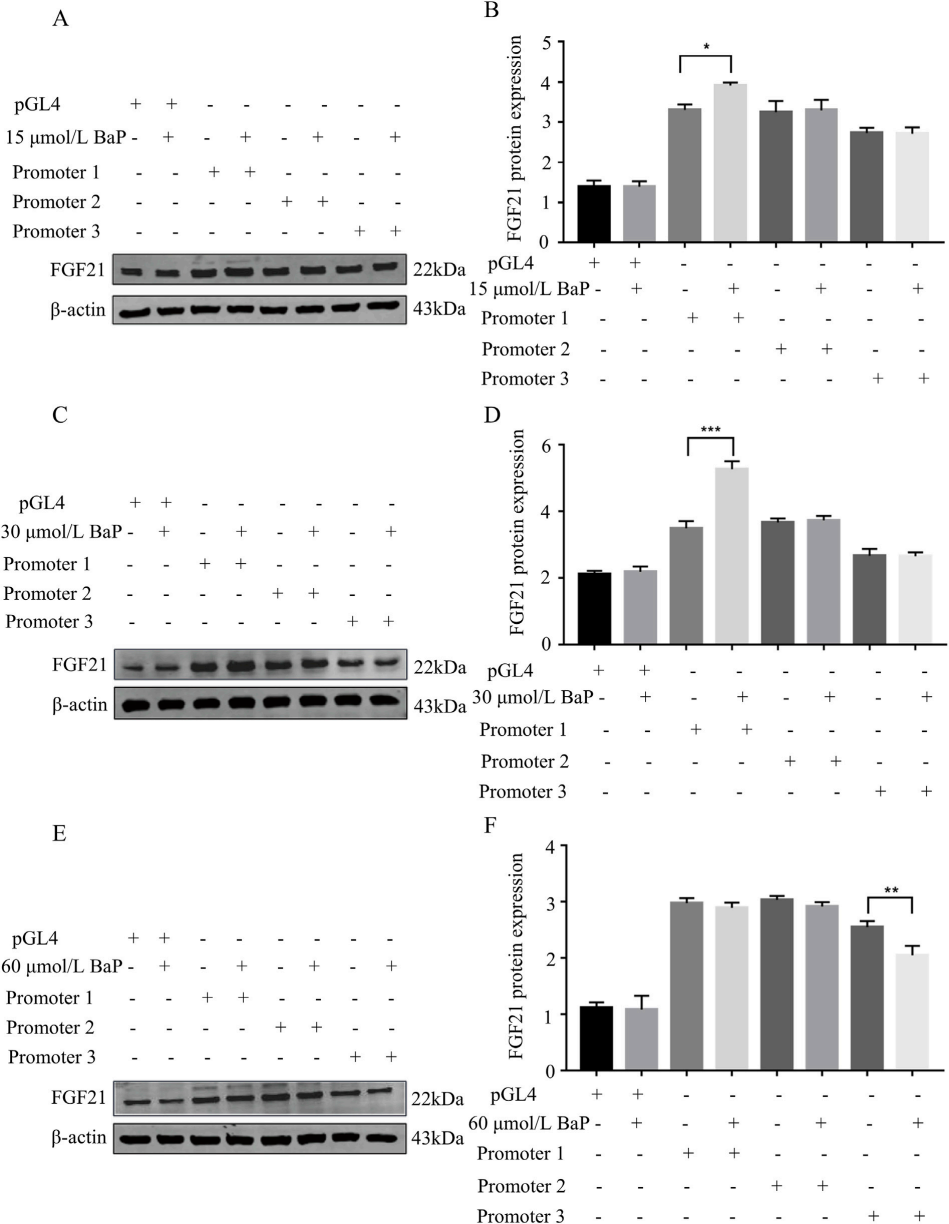
Effects of different concentrations of BaP on FGF21 protein expression in HepG2 cells transfected with recombinant plasmids (**(A)** 15 μmol/L BaP Protein bar graph, **(B)** Protein expression statistics, **(C)** 30 μmol/L BaP Protein bar graph, **(D)** Protein expression statistics, **(E)** 60 μmol/L BaP Protein bar graph, **(F)** Protein expression statistics, ^**^ *P* < 0.01 compared to transfected Promoter1, n = 3).

After transfecting HepG2 cells with recombinant plasmids, different concentrations of BaP were added, and FGF21 protein expression was measured in each group. Figures 9A,B show that compared to the promoter1 group, the promoter1 + 15 μmol/L BaP treatment significantly increased FGF21 protein expression. Similarly, Figures 9C,D illustrate that the promoter1 + 30 μmol/L BaP group exhibited a significant upregulation of FGF21 protein compared to the promoter1 group alone. In contrast, Figures 9E,F reveal that in the promoter3 + 60 μmol/L BaP group, FGF21 protein expression was significantly reduced compared to the promoter3 group. All these differences were statistically significant (*P* < 0.01). These results indicate that low concentrations of BaP enhance FGF21 expression through the promoter1 region, while high concentrations of BaP suppress FGF21 expression via the promoter3 region.

## Discussion

With the increasing prevalence of high-fat diets, unhealthy lifestyle habits, and industrial pollution, the incidence of liver-related disorders due to disrupted lipid metabolism has been steadily rising. Over the past two decades, the incidence rates of hepatic lipid metabolism disorders such as hepatitis, liver fibrosis, and non-alcoholic fatty liver disease (NAFLD) have significantly increased. Furthermore, the burden of progressive NAFLD-related diseases, such as non-alcoholic steatohepatitis (NASH), liver cirrhosis, and hepatocellular carcinoma, is expected to rise substantially. Therefore, preventing liver lipid metabolism-related diseases requires comprehensive and multi-faceted approaches. Cigarette smoke, industrial emissions, and charred foods impose considerable stress on hepatic metabolism, with polycyclic aromatic hydrocarbons (PAHs) being particularly harmful. In recent years, research on the relationship between PAHs and metabolic diseases has gained significant attention. For instance, Li et al. (2022) identified a strong positive correlation between PAH exposure and NAFLD incidence by studying 127 environmental pollutants. Additionally, our previous research demonstrated that continuous low-dose exposure to benzo[a]pyrene (BaP) for 12 weeks resulted in liver tissue damage, increased lipid accumulation, and fibrosis in mice (Lou et al., 2022). The primary routes of human PAH exposure are through air pollutants and cigarette smoke. To evaluate the toxic effects of environmental exposure on the liver, intratracheal instillation of BaP was performed in C57BL/6 mice, revealing that BaP exposure significantly increased the levels of glycerophospholipids and fatty acids in the liver (Li et al., 2019). These findings suggest that BaP ingestion may lead to hepatic lipid metabolic disorders and increased lipid accumulation, thereby contributing to NAFLD and other related diseases.

FGF21 is an important regulator of glucose and lipid metabolism discovered in recent years, which is mainly expressed in the liver and acts systemically after entering the blood circulation system. It has been found that FGF21 is associated with a variety of diseases related to glucose and lipid metabolism, such as obesity, fatty liver, coronary heart disease, atherosclerosis, myocardial infarction, etc (Zhang et al., 2021). It can affect the body through multiple targets and pathways. FGF21 can affect the level of glucose and lipid metabolism through multiple targets and pathways, and mainly regulates the level of lipid metabolism by affecting fatty acid β-oxidation and lipid synthesis-related factors. FGF21 can regulate fatty acid β-oxidation and mitochondrial function by activating PPARα, PGC-1α, CPT1α and other genes, and this interaction can help to maintain the energy balance in the body and participate in the regulation of glucose and lipid metabolism (Fisher and Maratos-Filer, 2016; Fisher et al., 2012; Nakamura et al., 2014; Zhou et al., 2019) PPARγ, C/EBPα and SCD1 are regulators closely related to lipid synthesis and play important roles in controlling lipid synthesis and adipocyte differentiation (Heikkinen et al., 2007; Yang et al., 2020; Cruz-Color et al., 2020). And there are also experiments that explored the chronic effects of FGF21 expression in mouse liver by injecting BaP, and found that BaP increased the expression of endogenous FGF21 in the subject animals (Shanehbandpour-Tabari.et al., 2024).

In our experiment, HepG2 cells were treated with 15, 30, and 60 μmol/L BaP, and lipid content in HepG2 cells was assessed using Nile Red fluorescence staining. The results showed that different concentrations of BaP led to varying degrees of lipid accumulation in HepG2 cells. BaP primarily exerts its effects through binding with the aryl hydrocarbon receptor (AhR) in the circulatory system, thereby activating AhR and its associated signaling pathways. AhR is predominantly expressed in the liver, and its downstream targets include several lipid metabolism-related factors such as PPARγ, PPARα, and CD38, which play crucial roles in hepatic lipid metabolism (Bock, 2019). Xia et al. (2019) identified AhR/CYP1A1 and AhR/TNF-α signaling pathways as contributors to insulin resistance and hepatic lipid accumulation. AhR’s impact on downstream genes is primarily mediated by its binding to xenobiotic response elements (XRE) within the promoter regions of target genes, which subsequently regulates gene expression (Sato et al., 2008). There are three XRE fragments in the FGF21 promoter region (Girer et al., 2019), suggesting that the AhR-DNA complex may regulate FGF21 expression, thereby influencing the expression of downstream lipid metabolism-related factors. Consequently, in this study, BaP and the AhR inhibitor CH223191 were used to observe the expression of FGF21 and related factors. The results demonstrated that BaP upregulated FGF21 and related factors in HepG2 cells through AhR, while AhR inhibition reduced the expression of FGF21 and related factors. This indicates that BaP’s effects on hepatic lipid metabolism are mediated through AhR.

AhR is involved in the transcriptional regulation of FGF21. Studies have shown that in C57BL/6 mice, FGF21 expression is dependent on the dose and duration of TCDD exposure. A single dose of 10 μg/kg or lower TCDD induced the most prominent increase in FGF21 mRNA expression at 6 h (Cheng et al., 2014). In contrast, when the TCDD dose exceeded 50 μg/kg, FGF21 mRNA levels increased in a time-dependent manner, peaking at 14 days and then declining. Girer et al. (2016) found that AhR agonists could reduce FGF21 expression, suggesting that multiple XREs exist within the FGF21 promoter region. When the AhR-ARNT heterodimer binds to specific XREs that overlap with the binding sites of lipid metabolism regulators such as PPARα, AhR agonists reduce FGF21 expression induced by PPARα and glucose in human liver cells. This indicates that AhR assists in maintaining hepatic energy balance by regulating FGF21 expression and its related signaling pathways. The above studies suggest that the AhR-ARNT heterodimer can bind to the FGF21 promoter and that AhR agonists can either activate or inhibit FGF21 transcription. Another study by Girer et al. (2019) showed that AhR agonists increased FGF21 gene expression when binding to XRE1 but decreased it when binding to XRE3. They speculated that under normal physiological conditions, AhR binding to XRE1 promoters transcriptional activation, while binding to XRE3 may inhibit FGF21 transcription in an agonist-dependent manner. However, further research is needed to validate these findings.

It remains unclear how BaP, a classical exogenous AhR ligand, affects FGF21 expression. In this experiment, different concentrations of BaP were applied to HepG2 cells, and we observed varying effects on FGF21 expression that did not follow a dose-dependent increase. This suggests that BaP at different concentrations may regulate the binding of AhR to different regions of the FGF21 promoter. To test this hypothesis, a dual-luciferase reporter gene assay was conducted. The results indicated that low-dose BaP may regulate AhR binding to XRE1 in the FGF21 promoter region, thereby increasing FGF21 expression, reducing hepatic lipid synthesis, and enhancing hepatic fatty acid β-oxidation. In contrast, high-dose BaP may regulate AhR binding to XRE3 in the FGF21 promoter region, leading to decreased FGF21 expression, increased hepatic lipid synthesis, and reduced hepatic fatty acid β-oxidation.

## Conclusion

Low-dose BaP increases the expression of FGF21, while high-dose BaP relatively reduces FGF21 expression. The effects of BaP on lipid metabolism in HepG2 cells are mediated through AhR activation. Low concentrations of BaP can enhance the activity of promoter regions containing the XRE1 sequence, thereby promoting FGF21 expression. In contrast, high concentrations of BaP inhibit the activity of promoter regions containing the XRE3 sequence, leading to a decrease in FGF21 expression. Through the above systematic study, the relationship between AhR and FGF-21 has been elucidated, which will provide experimental basis for the search of new targets for the prevention and treatment of NAFLD.

## Data Availability

The raw data supporting the conclusions of this article will be made available by the authors, without undue reservation.
